# Genome Sequencing of Wastewater Confirms the Arrival of the SARS-CoV-2 Omicron Variant at Frankfurt Airport but Limited Spread in the City of Frankfurt, Germany, in November 2021

**DOI:** 10.1128/MRA.01229-21

**Published:** 2022-01-27

**Authors:** Shelesh Agrawal, Laura Orschler, Simona Tavazzi, Robert Greither, Bernd Manfred Gawlik, Susanne Lackner

**Affiliations:** a Technical University of Darmstadt, Department of Civil and Environmental Engineering Sciences, Institute IWAR, Chair of Water and Environmental Biotechnology, Darmstadt, Germany; b European Commission, Joint Research Centre, Ispra, Varese, Italy; c Life Technologies, Darmstadt, Germany; DOE Joint Genome Institute

## Abstract

Wastewater-based severe acute respiratory syndrome coronavirus 2 (SARS-CoV-2) surveillance of Frankfurt Airport by genome sequencing was used to detect SARS-CoV-2 variants entering the region. In November 2021, we found all characteristic mutations of Omicron in wastewater originating from Frankfurt Airport before the first confirmed clinical report from an arriving passenger on 26 November 2021.

## ANNOUNCEMENT

Since the initial report from South Africa of the new severe acute respiratory syndrome coronavirus 2 (SARS-CoV-2) (genus *Betacoronavirus*, family *Coronaviridae*) variant that is known as the Omicron variant (WHO nomenclature) (1) or B.1.1.529 (PANGO lineage) (2), it has already been detected in 13 European Union and European Economic Area (EU/EEA) countries (3), most often from confirmed cases with a history of travel to southern African countries. The first positive report of Omicron from Frankfurt, Germany, was on 29 November 2021; the sample was collected on 26 November 2021 from a traveler who had arrived at Frankfurt Airport. In this work, we aimed to assess the earliest import of Omicron in Frankfurt by monitoring two wastewater streams, one partly originating from Frankfurt Airport and the other from the City of Frankfurt, using sequencing analysis. Using genomic sequencing could help overcome current limitations in detecting Omicron. Accordingly, the European Centre for Disease Prevention and Control (ECDC) suggested using genomic sequencing wastewater-based epidemiology (WBE) surveillance from incoming flights to track the import of Omicron (4).

For WBE surveillance of SARS-CoV-2 variants in wastewaters from Frankfurt Airport and Frankfurt City, we collected 24-h composite samples on 2 November and 23 November 2021. The samples were (i) sewage samples from a canal receiving wastewater from Frankfurt Airport and (ii) influent samples from the wastewater treatment plant (WWTP) of Frankfurt City. For sequencing, 500 mL of the untreated wastewater was concentrated by ultrafiltration with 100-kDa Centricon Plus-70 centrifugal ultrafilters (Merck), and RNA was extracted using the MagMAX Microbiome Ultra Nucleic Acid isolation kit (Thermo Fisher Scientific) according to the manufacturer’s protocol. Library preparation was performed according to a previous study (5). Briefly, the library was prepared using the Ion AmpliSeq SARS-CoV-2 research panel (Thermo Fisher Scientific) according to the manufacturer’s instructions. This panel consists of 237 primer pairs, resulting in an amplicon length range of 125 to 275 bp, which cover the nearly full genome of SARS-CoV-2. Libraries were multiplexed and sequenced using an Ion Torrent 540 chip on an Ion S5 sequencer (Thermo Fisher Scientific).

For data analysis, we used the directly installed software packages in Ion Torrent Suite v5.12.2 of our Ion S5 sequencer. We determined the genome coverage of our samples by mapping them to a SARS-CoV-2 reference genome (Wuhan-Hu-1 [GenBank accession numbers NC_045512 and MN908947.3]), using TMAP software included in the Ion Torrent Suite, after the unaligned reads were filtered using default parameters. The sequencing data for the samples are summarized in Table 1. For identification of mutations, all single-nucleotide variants (SNVs) were called using Variant Caller v5.16.0.5 with default parameters.

**TABLE 1:** Summary of the sequencing data for the samples

Sample location and collection date (day-mo-yr)	Total no. of reads	No. of mapped reads	Avg target base coverage depth (×)	Avg read identity vs target (%)^*a*^	GC content (%)	BioSample accession no.	SRA accession no.
Airport							
4-11-2021	1,169,001	345,149	319.5	95.61	62.9	SAMN24156791	SRR17258655
23-11-2021	8,299,055	3,245,669	9,693	98.36	55.8	SAMN24156792	SRR17258654
City							
4-11-2021	1,972,935	620,663	753.4	98.72	62.2	SAMN24156793	SRR17258653
23-11-2021	9,908,030	3,825,450	11,834	98.02	56.8	SAMN24156794	SRR17258652

a The target sequence was the SARS-CoV-2 reference genome (Wuhan-Hu-1 [GenBank accession numbers NC_045512 and MN908947.3]).

**FIG 1:**
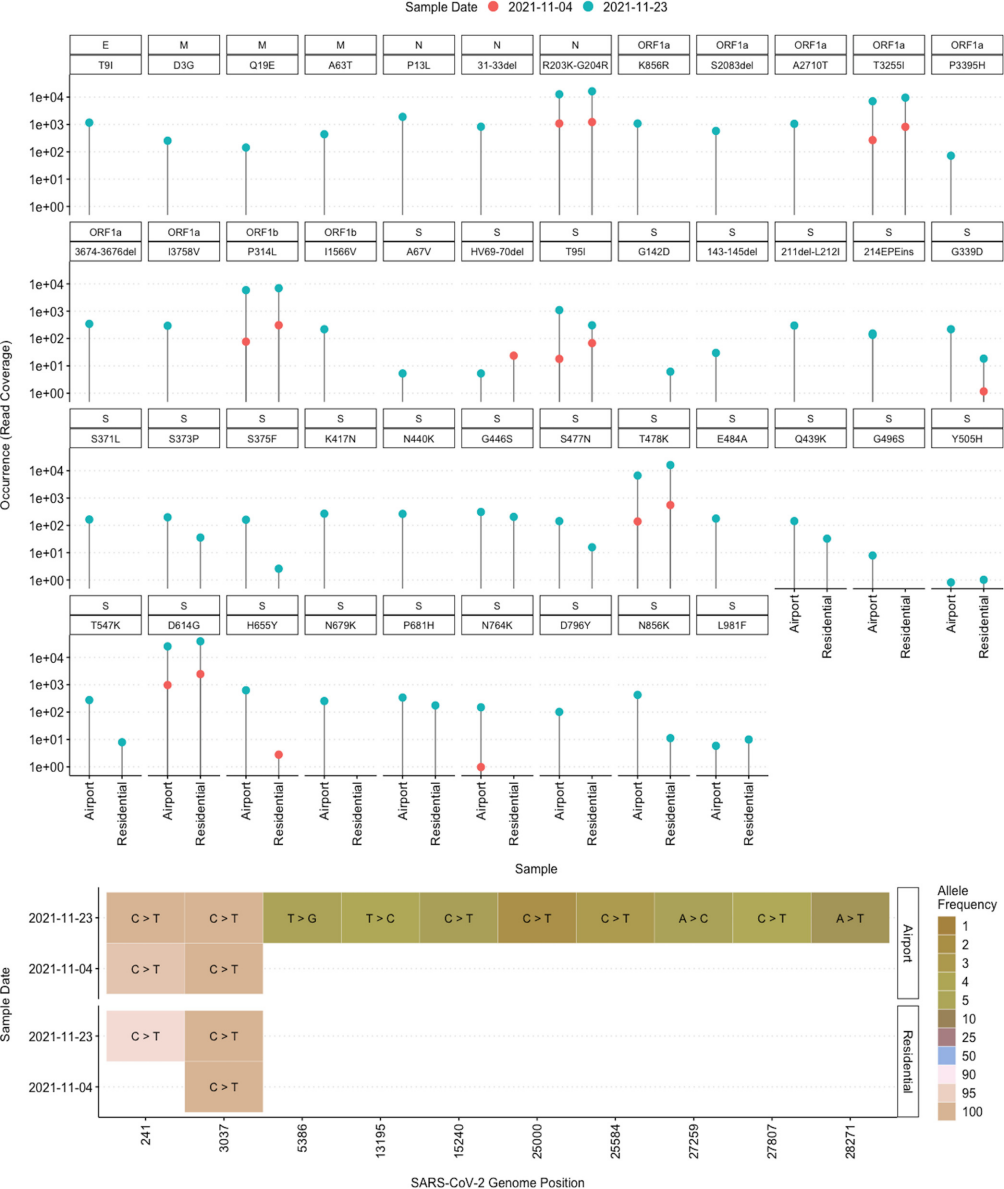
(Top) Occurrence (i.e., the numbers of reads corresponding to each mutation) of characteristic S, E, M, and N protein mutations and ORF1ab mutations of the Omicron variant. (Bottom) Heatmap showing the allele frequency and the alternate nucleotide of the characteristic synonymous mutations of the Omicron variant. Airport, wastewater sample with a significant fraction from Frankfurt Airport; Residential, influent wastewater sample collected from the WWTP of Frankfurt City.

We determined the numbers of reads corresponding to each of the characteristic mutations of the Omicron variant to estimate its prevalence in the samples. The Omicron variant has several mutations, compared to the reference Wuhan SARS-CoV-2 genome, including 37 mutations in the spike (S) protein, 3 mutations in the nucleocapsid (N) protein, 1 mutation in the envelope (E) protein, 3 mutations in the membrane (M) protein, and 10 synonymous mutations (6). Overall, we found the following S protein mutations in Omicron: A67V, HV69–70del, T95I, G142D, 143–145del, 211del-L212I, 214EPEins, G339D, S371L, S373P, S375F, K417N, N440K, G446S, S477N, T478K, E484A, Q439K, G496S, Y505H, T547K, D614G, H655Y, N679K, P681H, N764K, D796Y, N856K, and L981F (Fig. 1). Also, N protein mutations, an E protein mutation, M protein mutations, and mutations in the open reading frame 1ab (ORF1ab) replication complex were found. However, all of the Omicron-specific amino acid changes were detected only in the airport wastewater sample from 23 November 2021. We show that the surveillance of wastewater from transportation hubs, such as airports, using sequencing can support tracking of the import of SARS-CoV-2 variants.

### Data availability.

The raw metagenomic sequence data are available in the NCBI Sequence Read Archive (SRA) under BioProject number PRJNA789814.
